# Hydroelectric energy conversion of waste flows through hydroelectronic drag

**DOI:** 10.1073/pnas.2411613121

**Published:** 2024-10-17

**Authors:** Baptiste Coquinot, Lydéric Bocquet, Nikita Kavokine

**Affiliations:** ^a^Laboratoire de Physique de l’École Normale Supérieure, Université PSL, CNRS, Sorbonne Université, Université Paris Cité, Paris 75005, France; ^b^Department of Molecular Spectroscopy, Max Planck Institute for Polymer Research, Mainz 55128, Germany; ^c^Center for Computational Quantum Physics, Flatiron Institute New York, NY 10010; ^d^The Quantum Plumbing Lab, Ecole Polytechnique Fédérale de Lausanne, Lausanne 1015, Switzerland

**Keywords:** nanofluidics, energy conversion, solid–liquid interface

## Abstract

Hydraulic energy is a key component of the global energy mix, yet there exists no practical way of harvesting it at small scales, from flows with low Reynolds number. This has triggered a search for alternative hydroelectric conversion methodologies, leading to unconventional proposals based on droplet triboelectricity, water evaporation, osmotic energy, or flow-induced ionic Coulomb drag. Yet, these approaches systematically rely on ions as intermediate charge carriers, limiting the achievable power density. Here, we predict that the kinetic energy of small-scale “waste” flows can be directly and efficiently converted into electricity thanks to the hydroelectronic drag effect, by which an ion-free liquid induces an electronic current in the solid wall along which it flows. This effect originates in the fluctuation-induced coupling between fluid motion and electron transport. We develop a nonequilibrium thermodynamic formalism to assess the efficiency of such hydroelectric energy conversion, dubbed hydronic energy. We find that hydronic energy conversion is analogous to thermoelectricity, with the efficiency being controlled by a dimensionless figure of merit. However, in contrast to its thermoelectric analogue, this figure of merit combines independently tunable parameters of the solid and the liquid, and can thus significantly exceed unity. Our findings suggest strategies for blue energy harvesting without electrochemistry, and for waste flow mitigation in membrane-based filtration processes.

## Significance

At large scales, where fluid dynamics are governed by inertia, a turbine can convert this inertia into electricity with an efficiency in excess of 90%. At the smaller scales where viscous effects dominate, turbines are no longer efficient, and no satisfactory alternative has been identified so far. Yet, significant amounts of energy are lost to viscous flows in membrane-based filtration and osmotic energy conversion. Here, we propose a physical principle for nanoscale hydroelectric energy conversion—dubbed “hydronic energy”—which exploits the recently discovered fluctuation-induced quantum friction phenomenon. We develop a theoretical framework to assess its efficiency, and demonstrate its pertinence for generating electricity from liquid flows in the viscous regime.

Since its invention by Hero of Alexandria two thousand years ago, the turbine has been the tool of choice for converting the kinetic energy of a fluid flow into useful work. It is now a mature technology, with the hydroelectric conversion efficiency of modern turbines reaching over 90% (1). However, turbines only function at macroscopic scales and with sufficiently fast flows. When the Reynolds number decreases below the turbulence transition, the turbine efficiency plummets (2, 3). Thus, from an energetic standpoint, low Reynolds number flows are “waste flows,” in the same way as temperatures below 100 ^°^C are low-grade waste heat, still out of reach in terms of industrial energy recovery. Waste flows systematically arise in membrane-based filtration processes, as they involve liquids flowing through nanoscale pores (4–7). The kinetic energy of these flows is either lost to friction with the pore walls or dumped with the concentrated feed solution, and harvesting it could mitigate the energetic cost of filtration. There also exist various strategies for converting industrial waste heat into liquid flow (8, 9). Despite the ubiquity of waste flows, they have so far been converted to useful work only with limited efficiency.

Existing strategies are based on a wide range of astute physical principles, such as droplet impacts and triboelectricity (10–13), water evaporation (14–17), or electrolyte flows along conductors (18–22), to name a few. Although diverse, these methodologies are rooted in the streaming of dissolved ions under the liquid flow, which—through the image charge effect—results in an electronic current in the contiguous conducting wall: an effect that has been termed “ionic Coulomb drag.” These routes led to the design of small-scale energy generators, with various applications. However, their power-density remains overall limited, as well as difficult to scale up. In particular, ion polarization effects at membranes, and the charge imbalance associated with ion separation, are strong limitations to any ion-based energy conversion process (23).

## Ion-Free Hydrodynamic Coulomb Drag

However, the use of ions as intermediate charge carriers to induce an electronic current is not necessarily a prerequisite. Indeed, we have shown recently (24) that it is possible to directly convert the kinetic energy of a flowing liquid into an electronic current within the solid wall—a phenomenon dubbed hydrodynamic Coulomb drag (24) or hydroelectronic drag. In the following, we adopt the latter denomination, to emphasize the distinction with the ionic Coulomb drag described above. At the root of hydroelectronic drag is the solid–liquid quantum friction phenomenon (25–29), where fluctuating interactions between the liquid and the solid result in momentum transfer from the liquid to the solid’s electrons. The rate of this momentum transfer is quantified by the quantum or hydroelectronic friction coefficient, expressed as (25)

[1] λhe=18π2∫0+∞qdq(ħq)∫0+∞d(ħω)kBT……qsinh2ħω2kBTIm[ge(q,ω)]Im[gh(q,ω)]|1−ge(q,ω)gh(q,ω)|2,

where the functions $g_{h/e}$ are the surface response functions of the fluid and electrons in the solid, respectively, describing their charge density fluctuations (25). Quantum friction accounts, in particular, for the anomalous water permeability of carbon nanotubes, in terms of the anomalously large quantum friction on multilayer carbon surfaces as compared to isolated graphene layers (25, 30).

Overall, a liquid flow can thus transfer momentum to the solid’s electrons, resulting in an electric current proportional to the flow velocity. This process requires neither dissolved ions in the liquid, nor a surface charge on the solid wall. It is thus a mechanism very different from ionic Coulomb drag, and rather analogous to the “condensed-matter” Coulomb drag between two solid-state conductors (31).

We expect hydroelectronic drag to be one of the mechanisms at play in the various experiments that have revealed, in one way or another, an interaction between liquid or ion flow and solid-state electronic current. Unfortunately, many of these experiments have only been carried out with ionic solutions (18, 20, 21, 32), making it difficult to disentangle the contributions of ionic and hydroelectronic drag. A few experiments so far have shown electric current generation by ion-free liquids at the mesoscopic scale (33, 34). While qualitatively in line with hydroelectronic drag theory, the results are difficult to assess quantitatively, as the experiments did not precisely control the interfacial flow velocity; moreover, they show nonlinear flow velocity dependence, which points to mesoscopic effects that cannot be understood at the scale of the interface. Recently, however, our own experiments at the micron scale (35) could be quantitatively explained by a phonon-mediated version of hydroelectronic drag.

For a well-chosen solid–liquid system, the intrinsic hydroelectronic drag may exceed the contribution of dissolved ions. Indeed, the number of electrons set in motion through ionic Coulomb drag cannot exceed the number of ionic charges at the solid–liquid interface. Conversely, the liquid’s charge fluctuations—dubbed “hydrons” (27, 36)—when biased by the hydrodynamic flow, may set in motion all of the conduction electrons within a certain skin depth from the surface (*SI Appendix*, section 3). According to Eq. **1**, this requires a frequency matching between the hydron modes and the solid’s electronic excitations. To emphasize the crucial role played by hydrons, we will refer to the electrical energy produced through hydroelectronic drag as “hydronic energy”.

Motivated by these promising qualitative features, we undertake in this Article to quantitatively assess the efficiency of hydronic energy conversion. To this end, we develop a nonequilibrium thermodynamic formalism for the solid–liquid interface, and derive a dimensionless hydronic figure of merit that controls the conversion efficiency. We find that, in practical cases, this figure of merit can significantly exceed unity, highlighting the potential of hydroelectronic drag as a physical principle for energy harvesting from waste flows.

## Modeling a Hydronic Generator

**Fig. 1.**
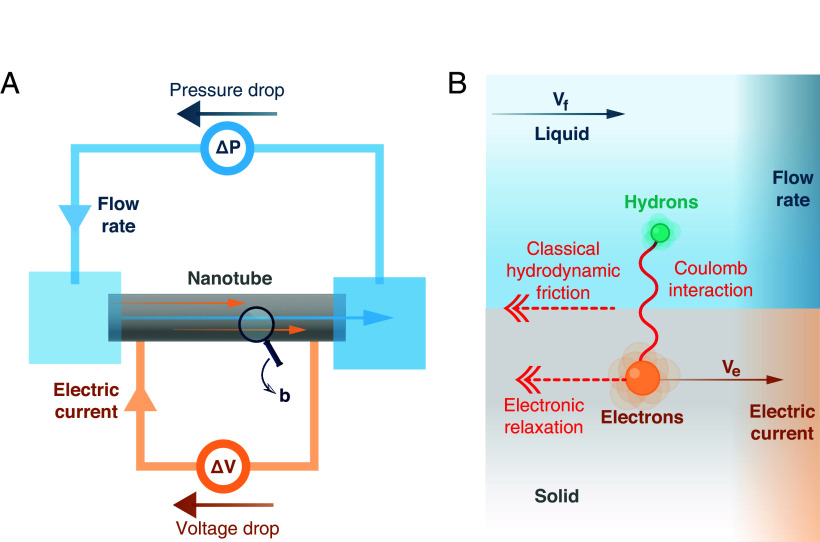
Coupled fluid and electron transport at the solid–liquid interface. (*A*) Sketch of a hydronic generator: A liquid flows through a nanotube, connected to an external electric circuit. Thanks to the hydroelectronic drag effect, an electric current is induced in the circuit in response to the liquid flow. (*B*) Schematic of the momentum transfer and relaxation processes at the solid–liquid interface, at the basis of hydroelectronic drag effect. Quantum (or hydroelectronic) friction transfers momentum directly from the liquid to the solid’s electrons. Momentum is relaxed at the interface through classical hydrodynamic friction, and inside the solid through electron scattering.

We consider the elementary building block of an energy conversion device based on hydroelectronic drag—which we dub “hydronic generator.” It consists in a nanoscale tube of length L, radius a, and thickness δ≪a, connected mechanically to two fluid reservoirs, and electrically to an external circuit that allows for electron circulation through the tube wall (Fig. 1). A pressure drop ΔP may be applied between the two reservoirs, and a voltage drop ΔV may be applied between the two solid-state electrical contacts. The fluid, with viscosity η, is assumed incompressible, and the Reynolds number is much smaller than 1, so that the flow rate through the tube in the absence of entrance effects is given by the Poiseuille law (37):

[2] $$Q=\frac{\pi a^{4}}{8\eta }\frac{\Delta P}{L}+\pi a^{2}v_{h}.$$

Here, we introduced a slip velocity $v_{h}$ for the fluid at the interface, which remains to be determined from the boundary conditions.

The electric current flowing through the solid wall can be rigorously determined from the electron’s nonequilibrium Green’s function renormalized by the water-electron Coulomb interactions, as was done in ref. 24 using Keldysh perturbation theory. Here, we use instead a simplified Drude model, which is quantitatively accurate for systems with a simple band structure (24), and has the advantage of being readily integrated into a thermodynamic formalism. The electric current is then $I=-2\pi a\delta \times n_{e}ev_{e}$, where $n_{e}$, where is the electron density and $v_{e}$ is the electron drift velocity, assumed uniform throughout the solid. In the Drude framework, we assume the electrons to have a parabolic dispersion with effective mass $m_{e}$ and a momentum-independent relaxation time $\tau_{e}^{0}$. A force balance on the electrons then yields

[3] $$I=\frac{n_{e}e^{2}\tau_{e}^{0}}{m_{e}}\Delta V-\frac{1}{L}\frac{e\tau_{e}^{0}}{m_{e}}F_{\mathrm{he}},$$

where $F_{\mathrm{he}}$ is the force exerted by the fluid on the electrons, which remains to be specified. In the following, we will use the notation $\lambda_{e}^{0}\equiv n_{e}m_{e}\delta /\tau_{e}^{0}$, a measure of electron relaxation that has the dimension of a friction coefficient. We emphasize again that the assumptions of the Drude model could be relaxed at this point (24), however at the expense of simplicity.

The flowing liquid transfers momentum to the channel wall through hydrodynamic friction. This momentum is redistributed between the wall’s various degrees of freedom (phonons, electrons) and is eventually relaxed to the environment. The total momentum flux (or force) $F_{\mathrm{hs}}$ from the liquid to the solid may be phenomenologically separated into two parts: a part that reaches the electrons (and is then dissipated by the electronic relaxation mechanisms) and a part that doesn’t. The latter corresponds to the classical (roughness-induced) friction (25). The former comprises hydroelectronic friction (Eq. **1**) and possibly a part due to phonons (24, 35, 38, 39). We will not consider the phonon contribution at this stage, and will thus provide lower bounds for the hydroelectronic coupling effects, in the absence of phononic enhancement. Quantitatively,

[4] $$F_{\mathrm{hs}}/\left(2\pi aL\right)=\lambda_{h}^{0}v_{h}+\lambda_{\mathrm{he}}\left(v_{h}-v_{e}\right),$$

where $\lambda_{h}^{0}$ is the classical friction coefficient and $\lambda_{\mathrm{he}}$ the hydroelectronic (quantum) friction coefficient, introduced in full generality in Eq. **1**. We then identify the hydroelectronic force as $F_{\mathrm{he}}=2\pi aL\lambda_{\mathrm{he}}\left(v_{h}-v_{e}\right)$. Finally, by enforcing global force balance on the liquid, $F_{\mathrm{hs}}=\pi a^{2}\Delta P$, we obtain a closed set of equations, that can be solved to obtain the “fluxes” Q and I as a function of the “forces” ΔP and ΔV.

## Hydroelectronic Transport Matrix

**Fig. 2.**
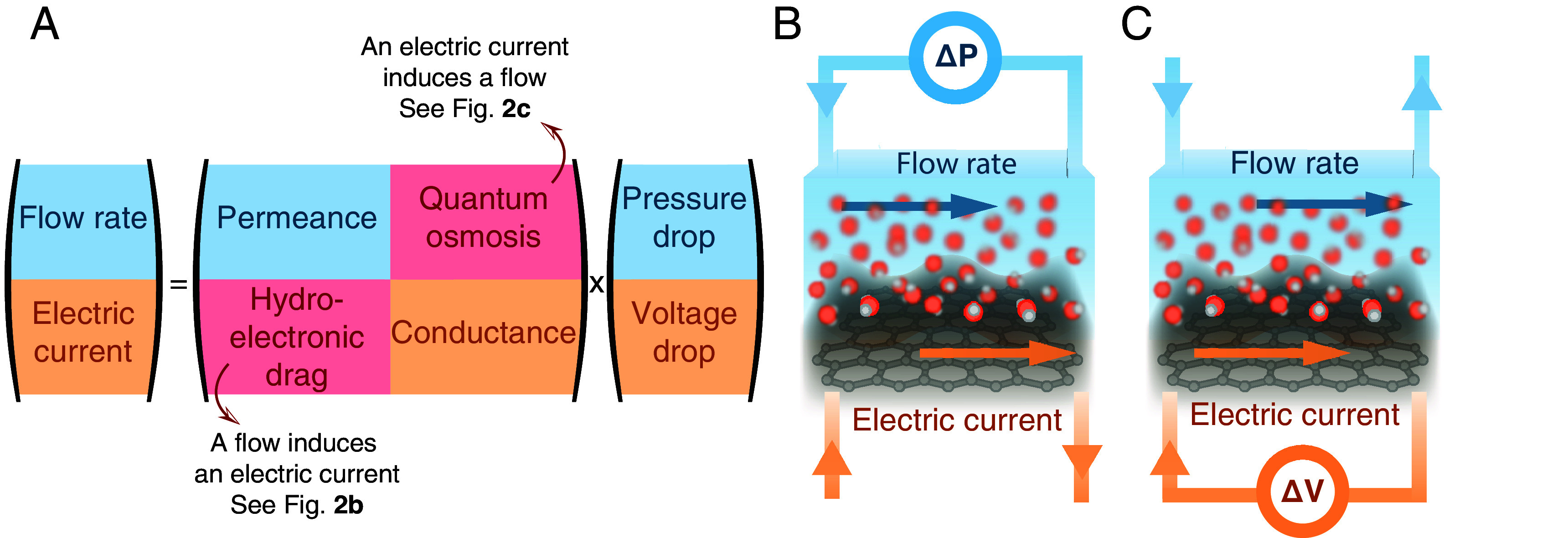
Transport matrix and hydroelectronic cross-couplings. (*A*) Sketch of the transport matrix of the system including a coupling (in red) between the flow sector (in blue) and the electronic sector (in orange). The two cross-terms correspond respectively to the hydroelectronic drag and quantum osmosis effects, and the corresponding transport coefficients are equal by Onsager’s symmetry. (*B*) Sketch of the hydroelectronic drag effect: A pressure drop generates a flow which induces an electric current through the hydroelectronic friction. (*C*) Sketch of the quantum osmosis effect: A voltage drop generates an electric current which induces a flow through the hydroelectronic friction.

The solution adopts a matrix structure (*SI Appendix*, section 1):

[5] $$\left(\begin{array}{c} Q\\ I \end{array}\right)=L\left(\begin{array}{c} \Delta P\\ \Delta V \end{array}\right)\;with\;L=\left(\begin{array}{cc} L & C_{\mathrm{he}}\\ C_{\mathrm{he}} & G \end{array}\right)$$

as sketched in Fig. 2*A*. The diagonal elements of the transport matrix are the permeance L and the conductance G. We find that the off-diagonal elements $C_{\mathrm{he}}$ are nonzero and equal, as expected from Onsager symmetry. Note that $C_{\mathrm{he}}$ is directly proportional to the “electrofluidic conductivity” introduced in ref. 24. Our model therefore not only predicts the hydroelectronic drag (a liquid flow induces an electric current, as sketched in Fig. 2*B*) but also its reciprocal effect (an electric current induces a liquid flow, as sketched in Fig. 2*C*), which has so far never been observed, and which we dub “quantum osmosis.” In the following, we will call $C_{\mathrm{he}}$ the “hydroelectronic mobility.”

To provide convenient expressions of the transport coefficients, we define a dimensionless hydroelectronic coupling constant

[6] $$\Lambda_{\mathrm{he}}=\frac{\lambda_{\mathrm{he}}^{2}}{\left(\lambda_{h}^{0}+\lambda_{\mathrm{he}}\right)\left(\lambda_{e}^{0}+\lambda_{\mathrm{he}}\right)}<1.$$

$\Lambda_{\mathrm{he}}$ quantifies the competition between liquid-electron momentum transfer ($\lambda_{\mathrm{he}}$) and the various sources of momentum relaxation: classical hydrodynamic friction ($\lambda_{h}^{0}$) and electron scattering ($\lambda_{e}^{0}$).

Accordingly, the expression for the hydroelectronic mobility $C_{\mathrm{he}}$ defined in Eq. **5**—at the core of the present study—takes the form

[7] $$C_{\mathrm{he}}=\frac{\pi a^{2}\delta }{L}\times \frac{en_{e}}{\lambda_{\mathrm{he}}}\times \frac{\Lambda_{\mathrm{he}}}{1-\Lambda_{\mathrm{he}}}.$$

It vanishes in the absence of hydroelectronic friction ($\Lambda_{\mathrm{he}}=0$) and grows to infinity as $\Lambda_{\mathrm{he}}\to$ 1.

The formalism also provides expression for the diagonal terms of the transport matrix: the permeance, which determines the flow rate Q under a pressure drop ΔP (for ΔV=0)

[8] $$L=\frac{\pi a^{4}}{8\eta L}\left(1+\frac{4b}{a(1-\Lambda_{\mathrm{he}})}\right),$$

where $b\equiv \eta /(\lambda_{\mathrm{ef}}+\lambda_{h}^{0})$ is the hydrodynamic slip length; and the conductance, which determines the electric current I under a voltage drop ΔV (for ΔP=0)

[9] $$G=\frac{1}{1-\Lambda_{\mathrm{he}}}\times \frac{2\pi a\delta }{L}\times \frac{e^{2}n_{e}\tau_{e}}{m_{e}},$$

where $\tau_{e}^{-1}=\left(\lambda_{e}^{0}+\lambda_{\mathrm{he}}\right)/\delta n_{e}m_{e}$ is the total electron scattering rate. These expressions highlight that the hydroelectronic coupling modifies the usual pressure-driven and voltage-driven transport phenomena. In the absence of hydroelectronic coupling ($\Lambda_{\mathrm{he}}=0$) the permeance and the conductance reduce to the Poiseuille formula and the Drude formula, respectively. When $\Lambda_{\mathrm{he}}\neq 0$, we find that both transport coefficients are enhanced. Physically, the flow-induced electric current boosts the liquid flow along the wall. Conversely, the current-induced liquid flow makes the electrons move faster inside the wall.

Overall, we have derived a transport matrix formalism for the solid–liquid interface, which bears analogy with the theoretical descriptions of osmotic effects in solution (40) or thermoelectric effects in the solid state (41, 42). We may check explicitly that the transport matrix L is definite positive, so that our model satisfies the second law of thermodynamics. We may also invert it in order to determine the voltage induced by the liquid flow in the case where the electric circuit is open, or the hydrostatic pressure induced by the electric current if the channel is closed (*SI Appendix*, section 1).

**Fig. 3.**
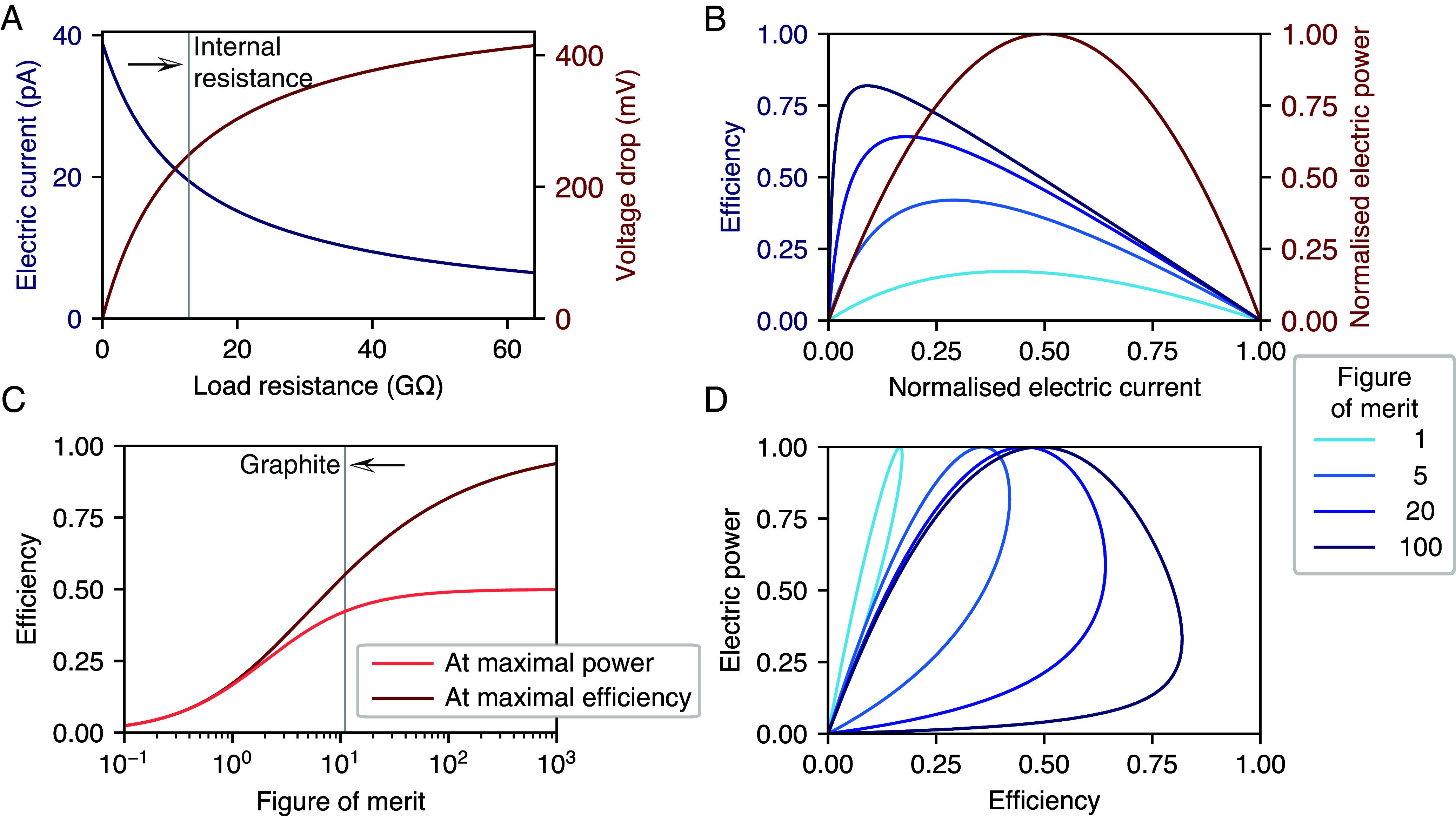
Energetics of the hydronic generator. (*A*) Open-circuit current and closed-circuit voltage produced in the hydronic generator as a function of the load resistance $R_{L}$. Here, we consider a single multiwall CNT with radius a=40nm and length L=10μm under a pressure drop of 1 bar. We use $n_{e}=2.3\cdot 10^{12}$ cm^−2^ for the electron density (25), and deduce an internal resistance $G^{-1}\approx 13$ GΩ for the nanotube, which is indicated by a vertical line. (*B*) Efficiency γ (in blue) and normalized electric power $P_{e}/P_{e}^{\max}$ (in red) as a function of the normalized electric current I. The efficiency is displayed for several values of the figure of merit Z; the normalized electric power is independent of the figure of merit. (*C*) Maximal efficiency $\gamma_{\max}$ and efficiency at maximal power (I=1/2) as a function of the figure of merit Z. The efficiency estimate for a graphite-based generator is indicated by a vertical line. (*D*) Efficiency-power diagram of the hydronic generator for different figures of merit Z. A loop-shaped curve is obtained as the load resistance is swept from zero to infinity.

At this point, we can provide a quantitative estimate of the hydroelectronic drag effect. As an example, we take a multiwall carbon nanotube of radius a=40nm, which was predicted to display significant hydroelectronic friction ($\lambda_{\mathrm{he}}/\lambda_{h}^{0}\approx 25$) (25). The closed-circuit current and open-circuit voltage across the tube under a pressure drop of 1 bar are displayed in Fig. 3*A* as a function of load resistance. At vanishing load resistance, the predicted electric current is 40 pA: The effect may thus be measurable at the scale of a single tube.

Let us now use the transport matrix to determine the efficiency of the hydronic generator.

## Efficiency and Figure of Merit

The mechanical power required to impose a flow rate Q through the hydronic generator is $P_{h}=Q\Delta P$. The transport matrix yields the corresponding pressure drop ΔP and the induced electric current I (*SI Appendix*, section 4). The resulting voltage drop is determined by the external load resistance $R_{L}$ connected to the electric circuit: $\Delta V=-R_{L}I$. The electric power delivered to the load resistance is then $P_{e}=-I\Delta V$, and the hydroelectric energy conversion efficiency is defined as $\gamma =P_{e}/P_{h}$.

The efficiency depends on the magnitude of the load resistance (to be compared with the internal resistance of the system $G^{-1}$), or equivalently on the electric current circulating through the system, as displayed in Fig. 3*B* (see also Fig. 3*A*). At vanishing load resistance, the maximum current is $I_{\max}=C_{\mathrm{he}}\Delta P$. For a given value of $R_{L}$, the efficiency can be expressed in terms of two dimensionless numbers, the normalized electric current $I=I/I_{\max}$ and the figure of merit Z:

[10] $$\gamma =\frac{I(1-I)}{I+1/Z},$$

with

[11] $$Z=\frac{\lambda_{\mathrm{he}}^{2}}{\lambda_{e}^{0}\lambda_{h}^{0}+\lambda_{\mathrm{he}}\left(\lambda_{e}^{0}+\lambda_{h}^{0}\right)}\frac{1}{1+(\lambda_{h}^{0}+\lambda_{\mathrm{he}})a/4\eta }.$$

This definition of the hydronic figure of merit is inspired by its thermoelectric analogue. Physically, Z compares the liquid-electron energy transfer rate ($\lambda_{\mathrm{he}}$) to the rate of energy dissipation in the system, which originates from electron scattering ($\lambda_{e}^{0}$), classical hydrodynamic friction ($\lambda_{h}^{0}$), and viscous effects in the fluid (η).

The efficiency vanishes when Z=0 and approaches 1 with increasing Z, as displayed in Fig. 3*C*: Here, the efficiency is not limited by the Carnot efficiency as there are no temperature gradients involved. For a given Z, there is a value of electric current I that achieves the maximum efficiency, given by

[12] $$\gamma_{\max}=\frac{Z}{{\left(1+\sqrt{1+Z}\right)}^{2}}=1-\frac{2}{\sqrt{Z}}+O\left(\frac{1}{Z}\right).$$

The delivered electric power is conveniently expressed as

[13] $$P_{e}=I\left(1-I\right)ZL^{0}{\left(\Delta P\right)}^{2},$$

where $L^{0}=\pi a^{4}\left(1+4b/a\right)/8\eta L$ is the permeance of the channel in the absence of flow-induced electric current. There is a value of I that achieves maximum power, which is different from the one that achieves maximum efficiency. This tradeoff is highlighted by the full power-efficiency diagram displayed in Fig. 3*D*.

We may now qualitatively analyze the requirements for a large figure of merit Z, and contrast them with the case of thermoelectricity. The thermoelectric figure of merit is essentially controlled by the ratio of electrical and thermal conductivity in a given material. These two quantities tend to vary together, which makes it difficult to achieve a figure of merit exceeding unity. In the hydroelectronic case, however, there is no contradiction between maximizing the electron–liquid interaction and minimizing dissipation (essentially, surface roughness and electron–phonon coupling). It is therefore of interest to make a quantitative assessment of the efficiency achievable in a hydronic generator and of the ensuing potential for waste flow recovery.

## Waste Flow Recovery

We first provide a simplified expression of the figure of merit by analyzing the relative importance of the dissipation mechanisms. Typical hydrodynamic friction coefficients are in the range $\lambda_{h}^{0}∼10^{3}\;\;\text{to}\;10^{6}$ Pa.s/m. The electron relaxation (mostly due to electron–phonon scattering at room temperature) is strongly material-dependent, but is typically in the range $\lambda_{e}^{0}∼10^{-2}\;\;\text{to}\;10^{3}$ Pa.s/m (*SI Appendix*, section 2). Thus, in most practical cases, dissipation is dominated by viscosity and interfacial friction effects and the figure of merit simplifies to

[14] $$Z\approx \frac{\lambda_{\mathrm{he}}}{\lambda_{h}^{0}}\times \frac{1}{1+a(\lambda_{\mathrm{he}}+\lambda_{h}^{0})/4\eta }.$$

This expression involves two physically meaningful quantities: the ratio of the hydroelectronic and classical friction coefficients and the ratio of channel radius and slip length $b=\eta /(\lambda_{\mathrm{he}}+\lambda_{h}^{0})$. The latter reflects that if the channel is large, most of the mechanical power is lost to viscous dissipation inside the fluid, rather than converted to electric power at the interface. Conversely, a sufficiently narrow channel (a≪b) allows for dissipation-free plug flow. The larger $\lambda_{\mathrm{he}}$, the narrower the channel needs to be to avoid viscous losses.

**Fig. 4.**
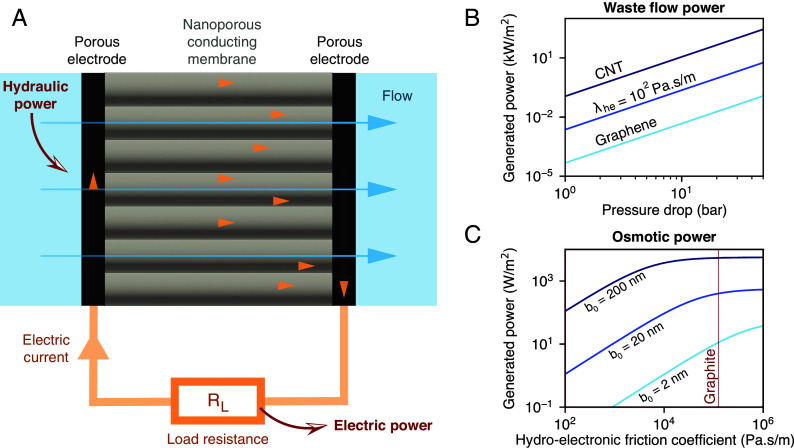
Membrane-scale hydronic generator for waste flow recovery. (*A*) Schematic of a hydronic generator based on a nanoporous conducting membrane placed between two porous electrodes. (*B*) Power recovered with a membrane-scale hydronic generator as a function of waste flow pressure, for different membrane materials. We consider multiwall CNTs with radius a=40nm, length L=10μm, and 50% packing fraction. We also consider pores with the same geometric and roughness characteristics, but with lower values of hydroelectronic friction coefficient: $\lambda_{\mathrm{he}}=10^{2}\;Pa.s/m$ and $\lambda_{\mathrm{he}}=1\;Pa.s/m$, as predicted for doped graphene (25). (*C*) Power produced by pressure-retarded osmosis through a semipermeable hydronic generator membrane. Here, the pores have a radius a=0.5 nm and 50% packing fraction. The power per unit membrane area is plotted as a function of the hydroelectronic friction coefficient ($\lambda_{\mathrm{he}}$), for different values of the classical friction coefficient ($b_{0}=\eta /\lambda_{h}^{0}$), and for a membrane thickness L=10μm. The curves flatten at large $\lambda_{\mathrm{he}}$ due to viscous dissipation in the membrane.

As a case study, let us analyze the performance of a hydronic generator based on a multiwall carbon nanotube (CNT) membrane (43, 44), as sketched in Fig. 4*A*. It was shown that, if sufficiently large, these tubes exhibit a specific plasmon mode, which results in significant quantum friction for water: $\lambda_{\mathrm{he}}/\lambda_{h}^{0}\approx 25$ for a tube of radius a=40nm (where a/(4b)≈1.3) (25, 30). The corresponding figure of merit is indeed significant: Z≈11, allowing for a maximum efficiency $\gamma_{\max}\approx 0.53$. Thus, a CNT-based hydronic generator could recover more than half of the energy lost to waste flows in a membrane-based filtration process. In reverse osmosis desalination, for example, sea water is pressurized to around 60 bar at the system inlet, and the brine released at the outlet is still at a high pressure, around 10bar. Large-scale desalination plants implement recovery devices for this brine flow, such as Pelton turbines and hydraulic pressure exchangers (45). While these devices work with efficiency close to unity in large plants, they are unsuitable for single-household or portable desalination systems. This is a technological gap that could potentially be filled by the hydronic generator. We note, however, that progress toward applications will require specific membrane development, beyond existing solutions. CNT membranes are indeed difficult to scale up, there exist many alternative materials that could exhibit similar properties [graphene oxides, lamellar conducting MXenes (46), etc.] and that should be considered as technological pathways to hydronic energy. As a guideline, materials with a relatively low density of high-effective-mass electrons are promising for achieving large figures of merit (*SI Appendix*, Fig. S1). For reference, Fig. 4*B* shows the recovered power per unit area as a function of waste flow pressure for a CNT membrane, as well as for model membrane materials characterized by their hydroelectronic friction coefficient.

A further promising application of the hydronic generator is blue energy harvesting through pressure retarded osmosis (PRO). In PRO, the osmotic pressure difference between fresh water and sea water—separated by a semipermeable membrane—is used to drive a water flow, which is fed into a turbine. Due to the low permeability of the membrane, a flow rate that is sufficient to spin a turbine is achieved only at the power plant scale. Replacing the turbine with a hydronic generator would make the technology viable at smaller scales: With the CNT-based model system, we estimate an achievable power density of $15\;W/m^{2}$ (*SI Appendix*, section 4), which is larger than what has so far been achieved with a PRO membrane and a turbine (<1 $W/m^{2}$) (7, 47). An even higher power density could be achieved with a hypothetical semipermeable and electrically conducting membrane, that would simultaneously express the osmotic pressure and harvest the energy from the resulting flow. Indeed, in PRO, most of the energy stored in the salinity gradient is lost to hydrodynamic friction with the membrane. A hydronic generator would instead convert this energy into electricity. With a semipermeable membrane that exhibits the same friction characteristics as graphite, a power density exceeding $1\;kW/m^{2}$ could be achieved (Fig. 4*C*). But even with moderate hydroelectronic friction ($10^{2}\;\;\text{to}\;10^{3}\;Pa.s/m$), a power density exceeding the industrial relevance threshold of $5\;W/m^{2}$ is within reach (7). We note that, in single nanopore systems, while ion-based osmotic power densities up to $1\;MW/m^{2}$ have been demonstrated (48, 49), this figure plummets upon scale-up due to concentration polarization effects and limitations of the electrodes (23). Here, the energy conversion mechanism is ion free; hence, it is expected to face fewer scale-up limitations. Therefore, developing selective membranes with low roughness and high conductivity may open a broad avenue toward blue energy harvesting.

## Perspectives

We have established several unique characteristics of hydroelectronic drag, that set it apart from other hydroelectric energy conversion principles. At the macroscopic scale, turbines are robust and efficient converters, based on inertial effects. As inertia vanishes at smaller scales, “chemical” energy conversion takes over: The energy stored in the fluid is first converted to an ionic current, which is then transformed into an electronic current thanks to an electrochemical reaction. Hydroelectronic drag uses neither inertia nor chemistry, and thus plays the role of a seemingly impossible “nanoscale turbine.”

We have developed a complete thermodynamic formalism for the hydroelectronic cross-couplings at the basis of this nanoscale turbine. A strong analogy can be established with thermoelectric cross-couplings: The induction of an electric current by a liquid flow is reminiscent of the Seebeck effect, while the induction of a liquid flow by an electric current (quantum osmosis) is analogous to the Peltier effect. But a key difference with thermoelectricity is that the relevant figure of merit is controlled by largely independent parameters and can therefore significantly exceed unity—a peculiarity rooted in the interfacial nature of hydroelectronic effects. Thanks to this peculiarity, hydroelectronic drag is a promising principle for harvesting energy from low Reynolds number waste flows, particularly those that arise in small-scale desalination and pressure-retarded osmosis. Technologies based on hydroelectronic drag will place constraints on membrane materials that are very different from those in electrochemical technologies, as the materials’ electronic excitations will come into play. Our results suggest that there are practical consequences for the water-energy nexus of the fundamental fact that, in nanofluidics, classical fluid dynamics meet the quantum dynamics of electronic matter.

## Supplementary Material

Appendix 01 (PDF)

## Data Availability

All data are included in the manuscript and/or *SI Appendix*.
