# Supplementary material for: Hydroelectric energy conversion of waste flows through hydroelectronic drag
Source: Proc Natl Acad Sci U S A. 2024 Oct 17;121(43):e2411613121. doi: 10.1073/pnas.2411613121 (PMC11513952; doi:10.1073/pnas.2411613121)
Supplement: Supplementary file 1 — Appendix 01 (PDF) [file pnas.2411613121.sapp.pdf]

PNAS

Supporting Information for

Hydroelectric energy conversion of waste flows through hydro-electronic drag

Baptiste Coquinot, Lydéric Bocquet[1], Nikita Kavokine[2]

[1]lyderic.bocquet@ens.fr [2]nkavokine@flatironinstitute.org

This PDF file includes:

Supporting text
Fig. S1
SI References

Supporting Information Text

1. Transport matrix

A. Computation of the transport matrix. The equation of motion for the liquid is:

$$\pi a^2 \times \Delta P = 2\pi a L \times [\lambda_{\mathrm{h}}^0 \mathbf{v}_{\mathrm{h}} + \lambda_{\mathrm{he}}(\mathbf{v}_{\mathrm{h}} - \mathbf{v}_{\mathrm{e}})]. \quad [1]$$

We denote $\lambda_{\mathrm{h}} = \lambda_{\mathrm{h}}^0 + \lambda_{\mathrm{he}}$ the total equilibrium friction coefficient. For the electrons, the equation of motion is

$$2\pi a d \times n_{\mathrm{e}} e \Delta V = 2\pi a L \times [\lambda_{\mathrm{e}}^0 \mathbf{v}_{\mathrm{e}} + \lambda_{\mathrm{he}}(\mathbf{v}_{\mathrm{e}} - \mathbf{v}_{\mathrm{h}})]. \quad [2]$$

where $\lambda_{\mathrm{e}}^0 = d n_{\mathrm{e}} m_{\mathrm{e}}/\tau_{\mathrm{e}}^0$ is the friction coefficient by surface area experienced by the electrons due to the electronic relaxation. We denote $\lambda_{\mathrm{e}} = \lambda_{\mathrm{e}}^0 + \lambda_{\mathrm{he}}$ the total equilibrium friction coefficient experienced by the electrons. It corresponds to a total electronic scattering time τ_{e} defined by $\lambda_{\mathrm{e}} = d n_{\mathrm{e}} m_{\mathrm{e}}/\tau_{\mathrm{e}}$.

These equations of motion have a common matrix structure given by

$$\begin{pmatrix} \lambda_{\mathrm{h}} & -\lambda_{\mathrm{he}} \\ -\lambda_{\mathrm{he}} & \lambda_{\mathrm{e}} \end{pmatrix} \begin{pmatrix} \mathbf{v}_{\mathrm{h}} \\ \mathbf{v}_{\mathrm{e}} \end{pmatrix} = \begin{pmatrix} a\Delta P/2L \\ e d n_{\mathrm{e}} \Delta V/L \end{pmatrix} \quad [3]$$

We need to invert this matrix to obtain the fluxes. The determinant of the matrix is

$$\det \begin{pmatrix} \lambda_{\mathrm{h}} & -\lambda_{\mathrm{he}} \\ -\lambda_{\mathrm{he}} & \lambda_{\mathrm{e}} \end{pmatrix} = \lambda_{\mathrm{h}}\lambda_{\mathrm{e}} - \lambda_{\mathrm{he}}^2 = \lambda_{\mathrm{h}}\lambda_{\mathrm{e}}(1 - \Lambda_{\mathrm{he}}) > 0 \quad [4]$$

where

$$\Lambda_{\mathrm{he}} = \frac{\lambda_{\mathrm{he}}^2}{\lambda_{\mathrm{h}}\lambda_{\mathrm{e}}} < 1 \quad [5]$$

is a dimensionless coefficient counting the efficiency of the solid-liquid coupling. Solving this linear system we obtain:

$$\begin{pmatrix} \mathbf{v}_{\mathrm{h}} \\ \mathbf{v}_{\mathrm{e}} \end{pmatrix} = \frac{1}{1 - \Lambda_{\mathrm{he}}} \begin{pmatrix} \lambda_{\mathrm{h}}^{-1} & \lambda_{\mathrm{he}}/(\lambda_{\mathrm{h}}\lambda_{\mathrm{e}}) \\ \lambda_{\mathrm{he}}/(\lambda_{\mathrm{h}}\lambda_{\mathrm{e}}) & \lambda_{\mathrm{e}}^{-1} \end{pmatrix} \begin{pmatrix} a\Delta P/2L \\ e d n_{\mathrm{e}} \Delta V/L \end{pmatrix} \quad [6]$$

The flow rate writes

$$Q = \frac{\pi a^4}{8\eta} \frac{\Delta P}{L} + \pi a^2 \mathbf{v}_{\mathrm{h}} \quad [7]$$

Thus, we deduce the permeance

$$\mathcal{L} = \frac{\pi a^4}{8\eta L} + \frac{1}{1 - \Lambda_{\mathrm{he}}} \frac{\pi a^3/L}{2(\lambda_{\mathrm{h}}^0 + \lambda_{\mathrm{he}})} = \frac{\pi a^4}{8\eta L}\left(1 + \frac{1}{1 - \Lambda_{\mathrm{he}}} \frac{4b}{a}\right) \quad [8]$$

where $b = \eta/(\lambda_{\mathrm{h}}^0 + \lambda_{\mathrm{he}})$ is the hydrodynamic slip length. We observe an effective slip length $b_{\mathrm{eff}} = b/(1 - \Lambda_{\mathrm{he}})$ in the absence of voltage drop, that is in a short circuit scenario. The slippage is larger because the electrons are in movement, leading to a cancelling of the hydro-electronic friction for the liquid. However, when the circuit is open, that is I is set to be zero, the slip length returns to b as no movement of electrons is allowed.

The electric current writes

$$I = 2\pi a d \times e n_{\mathrm{e}} \mathbf{v}_{\mathrm{e}} \quad [9]$$

Thus, the conductance is

$$G = \frac{1}{1 - \Lambda_{\mathrm{he}}} \frac{2\pi a (e d n_{\mathrm{e}})^2/L}{\lambda_{\mathrm{e}}^0 + \lambda_{\mathrm{he}}} = \frac{1}{1 - \Lambda_{\mathrm{he}}} \times \frac{2\pi a}{L} \frac{e^2 d n_{\mathrm{e}} \tau_{\mathrm{e}}}{m_{\mathrm{e}}} \quad [10]$$

Finally, the cross-term is

$$\mathcal{C}_{\mathrm{he}} = \frac{\pi a^2}{L} e d n_{\mathrm{e}} \frac{1}{1 - \Lambda_{\mathrm{he}}} \frac{\lambda_{\mathrm{he}}}{(\lambda_{\mathrm{h}}^0 + \lambda_{\mathrm{he}})(\lambda_{\mathrm{e}}^0 + \lambda_{\mathrm{he}})}. \quad [11]$$

Putting everything together, we have obtained:

$$\begin{pmatrix} Q \\ I \end{pmatrix} = \mathbf{L} \begin{pmatrix} \Delta P \\ \Delta V \end{pmatrix} \quad \text{with} \quad \mathbf{L} = \begin{pmatrix} \mathcal{L} & \mathcal{C}_{\mathrm{he}} \\ \mathcal{C}_{\mathrm{he}} & G \end{pmatrix}. \quad [12]$$

For later applications, let us provide the identities

$$\mathcal{C}_{\mathrm{he}} = \frac{a}{2 e d n_{\mathrm{e}}} \frac{\lambda_{\mathrm{he}}}{\lambda_{\mathrm{h}}^0 + \lambda_{\mathrm{he}}} G = \frac{2 e d n_{\mathrm{e}}}{a} \frac{\lambda_{\mathrm{he}}}{\lambda_{\mathrm{e}}^0 + \lambda_{\mathrm{he}}} \frac{\mathcal{L}}{1 + a/4b_{\mathrm{eff}}} \quad [13]$$

and

$$\mathcal{C}_{\mathrm{he}}^2 = \frac{\Lambda_{\mathrm{he}}}{1 + a/4b_{\mathrm{eff}}} \mathcal{L} G. \quad [14]$$

In particular, the determinant of the transport matrix is

$$\det(\mathbf{L}) = \mathcal{L} G \left(1 - \frac{\Lambda_{\mathrm{he}}}{1 + a/4b_{\mathrm{eff}}}\right) > 0 \quad [15]$$

which is indeed positive. Therefore, the matrix $\mathbf{L}$ is positive and symmetric.

B. Computation of the drag resistance. We now consider the case where we generate a flow rate Q through a pressure drop ΔP, which induces a voltage drop ΔV when the electric circuit is open, *i.e.* $I = 0$. Thus, the transport equations writes

$$\begin{pmatrix} Q \\ 0 \end{pmatrix} = \begin{pmatrix} \mathcal{L} & \mathcal{C}_{\rm he} \\ \mathcal{C}_{\rm he} & G \end{pmatrix} \begin{pmatrix} \Delta P \\ \Delta V \end{pmatrix}. \quad [16]$$

The inverse of the transport matrix writes

$$\mathbf{L}^{-1} = \frac{1}{1 - \frac{\Lambda_{\rm he}}{1+a/4b_{\rm eff}}} \begin{pmatrix} \mathcal{L}^{-1} & -\mathcal{C}_{\rm he}^{-1}\frac{\Lambda_{\rm he}}{1+a/4b_{\rm eff}} \\ -\mathcal{C}_{\rm he}^{-1}\frac{\Lambda_{\rm he}}{1+a/4b_{\rm eff}} & G^{-1} \end{pmatrix}. \quad [17]$$

As a consequence, the drag resistance defined by $\Delta V = -R_{\rm D} Q$ is

$$R_{\rm D} = \frac{L\Lambda_{\rm he}}{1 + a/4b_{\rm eff} - \Lambda_{\rm he}} \mathcal{C}_{\rm he}^{-1} \quad [18]$$

Using Eq. (13), we get

$$R_{\rm D} = \frac{L}{\pi a^2 e d n_{\rm e}} \frac{1 - \Lambda_{\rm he}}{1 + a/4b_{\rm eff} - \Lambda_{\rm he}} \lambda_{\rm he} \quad [19]$$

Finally, using that $b = \eta/(\lambda_{\rm h}^0 + \lambda_{\rm he})(1 - \Lambda_{\rm he})$, we deduce

$$R_{\rm D} = \frac{L}{\pi a^2 e d n_{\rm e}} \frac{\lambda_{\rm he}}{1 + (\lambda_{\rm h}^0 + \lambda_{\rm he})a/4\eta}. \quad [20]$$

Lastly, let us notice that the computation is identical when inverting the electric current with the flow rate by symmetry of the transport matrix. Thus, for a scenario where the liquid cannot flow, an electric current induces a pressure drop $\Delta P = -R_{\rm D} I$.

2. Materials

A. Jellium model. A jellium model is made of free electrons with an effective mass $m = m^* m_{\rm e}$ where $m_{\rm e}$ is the electron mass with a positive background (1). It is a standard model for the electronic properties of metals and semi-conductors. The energy is $E(k) = \hbar^2 k^2/2m$ and the effective mass tunes the band curvature. The electronic density $n_{\rm e}$ fixes the Fermi energy $E_{\rm F} = (\hbar^2/2m) \times (3\pi^2 n_{\rm e})^{2/3}$. To keep computations tractable, we consider the jellium at zero temperature, keeping in mind that the results will be qualitative rather than quantitative when the Fermi energy is comparable to temperature (2). Globally, we observe that the hydro-electronic friction coefficient becomes larger with larger effective mass and lower electronic density.

The electronic relaxation is $\lambda_{\rm e}^0 = d n_{\rm e} m/\tau_e^0$ where τ_e^0 is the electronic scattering time. As an order of magnitude, we will take $\tau_e^0 \sim 0.1$ ps, which is typical for graphene (3). Using a thickness $d = 1$ nm, we obtain

$$\lambda_{\rm e}^0 \sim 10 \times m^* \times n_{\rm e} \quad [n_{\rm e} \text{ in } /\text{nm}^3] \quad [21]$$

Thus, the effective mass and the electronic density should not be too high in order to avoid the electronic relaxation to become important.

The hydro-electronic friction coefficient, electronic relaxation coefficient and figure of merit (see Sec. IV) for a jellium model are given in Fig. 1. The best candidates are metallic systems with moderate electronic density and flat bands (high effective mass). For example, a jellium model with an electronic density $n_{\rm e} \sim 10^{26}/\text{m}^3$ and an effective mass $m^* = 100$ would have an hydro-electronic friction coefficient $\lambda_{\rm he} \approx 2 \cdot 10^2$ Pa.s/m and an electronic relaxation $\lambda_{\rm e}^0 \approx 1 \cdot 10^2$ Pa.s/m $\ll \lambda_{\rm he}$. Assuming a smooth solid with slip length $b_0 \approx 200$ nm like on graphene and no viscous dissipation ($a \ll b$), this corresponds to a figure of merit of $Z \approx 4 \cdot 10^{-2}$. If the effective mass is boosted to $m^* = 1000$, the hydro-electronic friction coefficient booms to $\lambda_{\rm he} \approx 6 \cdot 10^3$ Pa.s/m with an electronic relaxation $\lambda_{\rm e}^0 \approx 1 \cdot 10^3$ Pa.s/m $\ll \lambda_{\rm he}$. This leads to a figure of merit of $Z \approx 1$.

B. Numerical values for different physical materials.

Graphene. The friction on atomically smooth graphene is weak. While there are large discrepancies in the literature for the slip length on graphene, we can use the conservative value of $b = 200$ nm. The hydro-electronic friction on graphene is very low. According to (2), for a carrier density of $n_{\rm e}^{2d} \approx 10^{14}/\text{cm}^2$, the hydro-electronic friction coefficient becomes $\lambda_{\rm he} \approx 1$ Pa.s/m. Thus, the friction of graphene is dominated by the roughness-induced friction: $\lambda_{\rm h}^0 \approx 5 \cdot 10^3$ Pa.s/m. The electronic mobility is graphene is high, typically $\mu \approx 2 \cdot 10^1$ C.s/kg. Thus, for a carrier density of $n_{\rm e}^{2d} \approx 10^{14}/\text{cm}^2$ (strongly doped graphene) we deduce $\lambda_{\rm e}^0 \approx 10^{-2}$ Pa.s/m. The corresponding figure of merit is $Z \approx 4 \cdot 10^{-4}$.

Graphite. The classical friction on atomically smooth graphite is the same than on graphene, thus $\lambda_{\rm h}^0 \approx 5 \cdot 10^3$ Pa.s/m. However, the slip length on graphite is measured to be $b = 8$ nm. This strong friction was associated to the the hydro-electronic coupling with the plasmon of graphite (2). We thus deduce $\lambda_{\rm he} \approx 1.3 \cdot 10^5$ Pa.s/m. At room temperature, the effective surface charge carrier density of graphite is estimated to $n_{\rm e}^{2d} \approx 2.3 \cdot 10^{12}/\text{cm}^2$. The in-plane conductivity of graphite is measured to be $\sigma \approx 2 \cdot 10^4$ C^2.s/kg.m^3. For a Drude model, $\sigma \approx n_{\rm e}^{2d} e^2 \tau_{\rm e}^0/\delta_{\rm il} m_{\rm e}$ where $\delta_{\rm il} \approx 0.335$ nm is the inter-layer distance. Thus, $\lambda_{\rm e}^0 \approx N_{\rm layer}(e n_{\rm e}^{2d})^2/\sigma\delta_{\rm il} \approx 2N_{\rm layer}$ Pa.s/m, which remains low even for a large number of layers $N_{\rm layer}$. For graphite, the figure of merit is $Z \approx 11$.

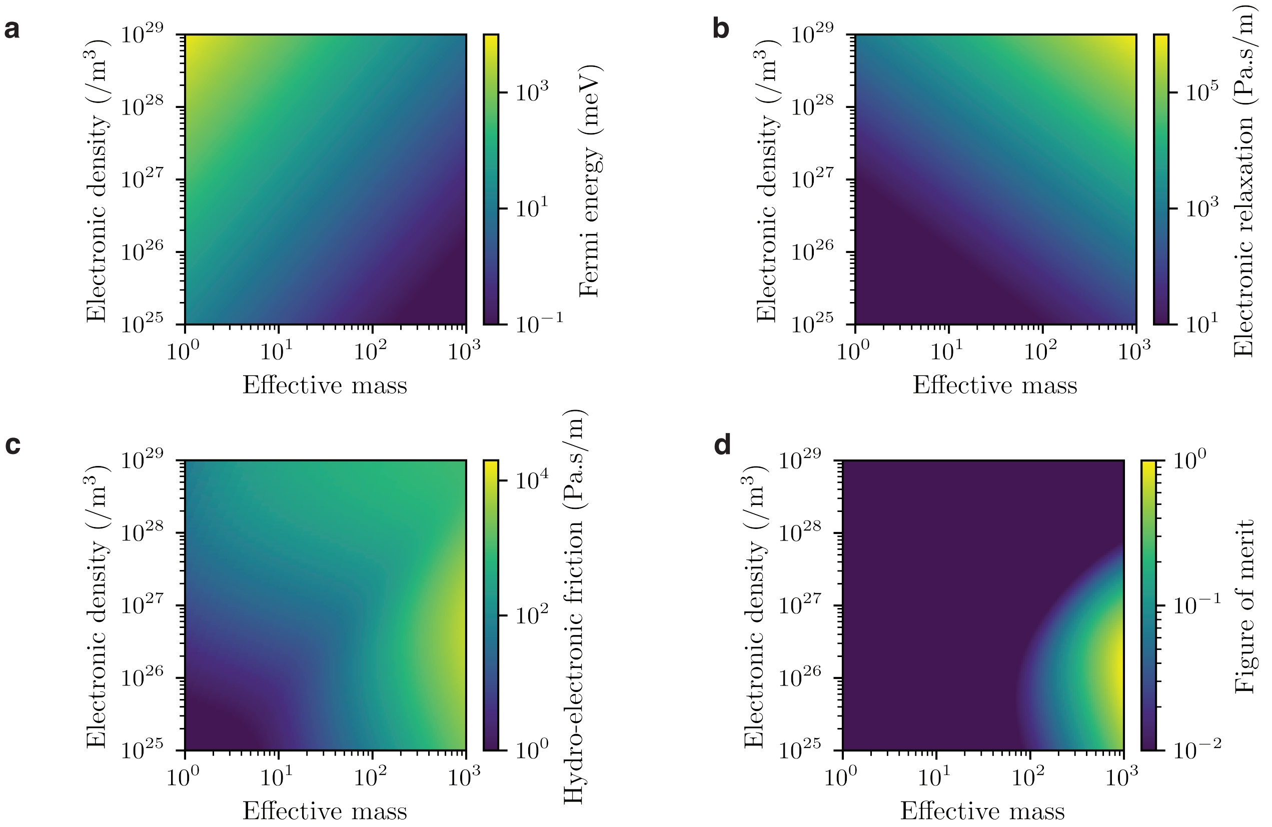

Fig. S1. Phase diagram of the Jellium model efficiency for the hydronic generator. The parameters are the effective mass m^* and the electronic density n_e. The jellium model is assumed to be at zero temperature for simplicity. **(a)** Effective Fermi energy of the model. **(b)** Electronic relaxation coefficient λ_e^0. **(c)** Hydro-electronic friction coefficient λ_he. **(d)** Associated figure of merit Z.

Copper. Assuming one electron per atom, the carrier density of copper is $n_\mathrm{e} \approx 10^{29}/\mathrm{m}^3$. The conductivity of clean copper is $\sigma \sim 6 \cdot 10^7$ C^2.s/kg.m^3. Thus, $\lambda_\mathrm{e}^0 \approx \delta (e n_\mathrm{e})^2/\sigma \approx 4 \cdot 10^3$ Pa.s/m for a thickness of 1 nm. For a jellium model (1), the effective mass of electrons is close to unity (4). The Fermi energy of a jellium model with the same electronic density is around 7 eV, close to the Fermi energy of copper. The resulting hydro-electronic friction, computed using the method of (2), is around $6 \cdot 10^1$ Pa.s/m.

Aluminium. Assuming one electron per atom, the carrier density of aluminium is $n_\mathrm{e} \approx 10^{29}/\mathrm{m}^3$. The conductivity of clean aluminium is $\sigma \sim 4 \cdot 10^7$ C^2.s/kg.m^3. Thus, $\lambda_\mathrm{e}^0 \approx \delta (e n_\mathrm{e})^2/\sigma \approx 6 \cdot 10^3$ Pa.s/m for a thickness of 1 nm. For a jellium model (1), the effective mass of electrons is close to unity (5). The Fermi energy of a jellium model with the same electronic density is around 7 eV, around half the Fermi energy of aluminium. The resulting hydro-electronic friction, computed using the method of (2), is around $6 \cdot 10^1$ Pa.s/m.

C. Numerical values for permeances.

Remark on units. The most common unit in the literature for the permeance is:

$$1 \ \mathrm{L/m^2.h.bar} \approx 2.78 \cdot 10^{-12} \ \mathrm{m/Pa.s} \qquad [22]$$

Selective membrane for pressure retarded osmosis (PRO). Commercial membranes selective to salt currently have a permeance of typically (6):

$$\mathcal{L}_h^* \sim 1 - 3 \ \mathrm{L/m^2.h.bar} \qquad [23]$$

Membrane of graphitic nanotubes. For a membrane of graphitic nanotubes of nanometric radius a, length $L \sim 10$ μm and pore density close to compact packing $\phi \sim 1/\pi a^2$, the permeance per area is

$$\mathcal{L}^* = \frac{\pi a^4 \phi}{8 \eta L} \left(1 + \frac{1}{1 - \Lambda_\mathrm{he}} \frac{4 b_\mathrm{graphite}}{a} \right) \approx \frac{a b_\mathrm{graphene}}{2 \eta L} \approx 10^{-8} \times a \ \mathrm{m/Pa.s} \ [a \text{ in nm}] \qquad [24]$$

where we have used $b_\mathrm{graphene} \approx 200$ nm (7).

3. Ionic Coulomb drag vs. Hydrodynamic Coulomb drag

Ionic Coulomb drag corresponds to an electronic current induced by the ionic streaming current in solution (8). In presence of a fixed surface charge Σ a flow of liquid at velocity $\mathbf{v}_\mathrm{h}$ will drag the counter-ions in the solution leading to an ionic current $\mathbf{j}_\mathrm{sc} = -\Sigma \mathbf{v}_\mathrm{h}$. If the solid is a metal or a semi-conductor, each of these charges has an image charge in the solid. The ionic current then reflects into an electronic current of the image charge, which cannot exceed $\mathbf{j}_\mathrm{i} = \Sigma \mathbf{v}_\mathrm{h}$. This is the ionic Coulomb drag when the electronic circuit is in short circuit, *i.e.* there is no electric field.

This current can be compared with the current generated by hydrodynamic Coulomb drag, which is based on the fluctuation-induced coupling between the liquid's dielectric fluctuations – called hydrons – and the electrons. Here, there is no ion. The electronic current in short circuit is $\mathbf{j}_\mathrm{h} = e n_\mathrm{e}^{2d} \mathbf{v}_\mathrm{e}$ where the 2d- electronic density is $n_\mathrm{e}^{2d} = n_\mathrm{e} \delta$ where δ is the skin length over which the electrons are dragged with the liquid. Using Eq. (6), the electronic velocity can be compared to the liquid's velocity as $\mathbf{v}_\mathrm{e} = \lambda_\mathrm{he}/(\lambda_\mathrm{e} + \lambda_\mathrm{he}) \times \mathbf{v}_\mathrm{h}$. Thus, the hydrodynamic Coulomb drag current writes

$$\mathbf{j}_\mathrm{h} = e n_\mathrm{e} \delta \frac{\lambda_\mathrm{he}}{\lambda_\mathrm{e} + \lambda_\mathrm{he}} \mathbf{v}_\mathrm{h} \qquad [25]$$

As expected, if the hydro-electronic coupling is weak compared with the electron's scattering, the hydrodynamic Coulomb drag is inefficient and the ionic Coulomb drag dominates. However, we have seen that on many materials of interest, the hydro-electronic coupling dominates the electron's scattering ($\lambda_\mathrm{e} \ll \lambda_\mathrm{he}$), in which case the electron's velocity $\mathbf{v}_\mathrm{e}$ is closed to the liquid's velocity $\mathbf{v}_\mathrm{h}$.

Thus, for material exhibiting a good coupling with the liquid's modes, both the liquid and the electrons move at the same velocity. Comparing the ionic to hydrodynamic Coulomb drag then reduce to compare the ionic and electronic densities of charge. Thus, the ratio can be written as an electronic Dukhin number $\mathrm{Du_e}$ defined by:

$$\frac{\mathbf{j}_\mathrm{i}}{\mathbf{j}_\mathrm{h}} \approx \frac{\Sigma}{e n_\mathrm{e} \delta} =: \mathrm{Du_e} \qquad [26]$$

Here δ represents the thickness of the solid wall or the penetration depth of the hydro-electronic drag, whichever is larger: we will take the conservative estimate $\delta \approx 1$ nm. Then, for a typical metal (1 electron per atom), we find $n_\mathrm{e} \delta \approx 10^3$ mol/L, so that $\mathrm{Du_e}$ is much smaller than 1 for all practical surface charges, and hydrodynamic Coulomb drag is the dominant effect. For a doped semiconductor ($n_\mathrm{e} \delta \approx 10^{16} - 10^{17}$ m^{-2}), we find that hydrodynamic Coulomb drag dominates if the surface charge is lower than $\Sigma_\mathrm{c} \sim 10$ mC/m^2.

4. Hydro-electronic generator efficiency

A. Efficiency under a pressure drop. We now generate a flow with a pressure drop and feed the electric current into a load resistance R_L. Thus, $\Delta V = -R_L I$. The transport equations now write

$$\begin{pmatrix} Q \\ I \end{pmatrix} = \begin{pmatrix} \mathcal{L} & \mathcal{C}_{\rm he} \\ \mathcal{C}_{\rm he} & G \end{pmatrix} \begin{pmatrix} \Delta P \\ -R_L I \end{pmatrix}. \qquad [27]$$

Thus, the current writes

$$I = \frac{I_{\max}}{1 + GR_L} \quad \text{with} \quad I_{\max} = \mathcal{C}_{\rm he}\Delta P \qquad [28]$$

ans saturates at small load resistance. Defining $\mathcal{I} = I/I_{\max}$ the normalised current we can use it as a free parameter instead of R_L. Indeed,

$$GR_L = \frac{1-\mathcal{I}}{\mathcal{I}}. \qquad [29]$$

The electric power delivered is

$$\mathcal{P}_{\rm e} = R_L I^2 = \frac{R_L}{(1+GR_L)^2} I_{\max}^2 \qquad [30]$$

It reaches a maximum for $R_L = 1/G$ and the maximal electric power is

$$\mathcal{P}_{\rm e}^{\max} = \frac{\mathcal{C}_{\rm he}^2}{G}\frac{(\Delta P)^2}{4} = \frac{\Lambda_{\rm he}}{1 + a/4b_{\rm eff}}\frac{\mathcal{L}(\Delta P)^2}{4} \qquad [31]$$

Using the definition of $\mathcal{L}$ in Eq. (8), the maximal electric power is

$$\mathcal{P}_{\rm e}^{\max} = \frac{\Lambda_{\rm he}}{1-\Lambda_{\rm he}}\frac{\pi a^3}{2(\lambda_{\rm h}^0 + \lambda_{\rm he})}\frac{(\Delta P)^2}{4L} \qquad [32]$$

Returning to the electric power expressed as a function of the parameter $\mathcal{I}$, it becomes

$$\frac{\mathcal{P}_{\rm e}}{\mathcal{P}_{\rm e}^{\max}} = 4\frac{GR_L}{(1+GR_L)^2} = 4\mathcal{I}(1-\mathcal{I}). \qquad [33]$$

which is the same equation than for an thermo-electric generator.

The flow rate is

$$Q = \mathcal{L}\Delta P - R_L\mathcal{C}_{\rm he} I = \left(1 - \frac{\Lambda_{\rm he}}{1 + a/4b_{\rm eff}}(1-\mathcal{I})\right)\mathcal{L}\Delta P \qquad [34]$$

The mechanical power delivered by the flow is

$$\mathcal{P}_{\rm h} = Q\Delta P = \mathcal{L}(\Delta P)^2 - \frac{R_L}{1+GR_L}\mathcal{C}_{\rm he}^2(\Delta P)^2 \qquad [35]$$

which can be written

$$\mathcal{P}_{\rm h} = \left(1 - \frac{GR_L}{1+GR_L}\frac{\Lambda_{\rm he}}{1 + a/4b_{\rm eff}}\right)\mathcal{L}(\Delta P)^2. \qquad [36]$$

Using the parameter $\mathcal{I}$, the mechanical power of the fluid writes

$$\mathcal{P}_{\rm h} = \left(1 - \frac{\Lambda_{\rm he}}{1 + a/4b_{\rm eff}}(1-\mathcal{I})\right)\mathcal{L}(\Delta P)^2. \qquad [37]$$

Thus, the efficiency of the hydronic generator is

$$\gamma = \frac{\mathcal{P}_{\rm e}}{\mathcal{P}_{\rm h}} = \Lambda_{\rm he}\frac{\mathcal{I}(1-\mathcal{I})}{1 + a/4b_{\rm eff} - \Lambda_{\rm he}(1-\mathcal{I})} \qquad [38]$$

Defining the figure of merit by

$$\mathrm{Z} = \frac{\Lambda_{\rm he}}{1 - \Lambda_{\rm he} + a/4b_{\rm eff}} = \frac{\Lambda_{\rm he}}{1-\Lambda_{\rm he}}\frac{1}{1 + (\lambda_{\rm e}^0 + \lambda_{\rm he})a/4\eta}, \qquad [39]$$

the efficiency becomes

$$\gamma = \frac{\mathcal{P}_{\rm e}}{\mathcal{P}_{\rm h}} = \frac{\mathcal{I}(1-\mathcal{I})}{\mathcal{I} + 1/Z}. \qquad [40]$$

This figure of merit is then the only parameter to control the efficiency. To better understand it, we can express it explicitly in term of friction coefficients.

$$\mathrm{Z} = \frac{\lambda_{\rm he}^2}{\lambda_{\rm e}^0\lambda_{\rm h}^0 + \lambda_{\rm he}(\lambda_{\rm e}^0 + \lambda_{\rm h}^0)}\frac{1}{1 + (\lambda_{\rm e}^0 + \lambda_{\rm he})a/4\eta}, \qquad [41]$$

It ranges from zero when the hydro-electronic coupling vanishes to infinity when the relaxation processes disappear.

The maximum of the efficiency is reached for $\mathcal{I} = 1/\sqrt{1+Z}$ and achieves

$$\gamma_{\max} = \frac{Z}{(1+\sqrt{1+Z})^2}. \quad [42]$$

When $Z \to \infty$, the efficiency goes to 1: the conversion is perfect. The electric power at maximal efficiency is

$$\frac{\mathcal{P}_{\mathrm{e}}}{\mathcal{P}_{\mathrm{e}}^{\max}} = 4\frac{\sqrt{1+Z}-1}{1+Z}. \quad [43]$$

The efficiency at maximal power is also a key information. The electric power is maximum for $\mathcal{I} = 1/2$ and the associated efficiency is

$$\gamma_{\mathrm{eff}} = \frac{1}{2}\frac{Z}{Z+2} = \frac{1}{2}\frac{\lambda_{\mathrm{he}}^2}{2\lambda_{\mathrm{e}}\lambda_{\mathrm{h}} - \lambda_{\mathrm{he}}^2}. \quad [44]$$

When $Z \to \infty$, the efficiency saturates to 1/2 but the maximal electric power explodes. Indeed,

$$\mathcal{P}_{\mathrm{e}}^{\max} = \frac{\Lambda_{\mathrm{he}}}{1+a/4b_{\mathrm{eff}}}\frac{\mathcal{L}(\Delta P)^2}{4} = Z\frac{\mathcal{L}^0(\Delta P)^2}{4} \quad [45]$$

where

$$\mathcal{L}^0 = \frac{\pi a^4}{8\eta L}\left(1+\frac{4b}{a}\right) \quad [46]$$

is the membrane's permeance in the absence of flow-induced electric current. In the meantime, the flow rate is:

$$Q = \left(1+\frac{Z}{2}\right)\mathcal{L}^0\Delta P \quad [47]$$

Thus, the electric power in terms of the flow rate writes:

$$\mathcal{P}_{\mathrm{e}}^{\max} = \frac{Z}{(Z+2)^2}\frac{Q^2}{\mathcal{L}^0} \quad [48]$$

B. Efficiency for membranes in series under a pressure drop. Let us now consider the case where the hydronic generator is a membrane of area $\mathcal{A}_{\mathrm{he}}$ and pore density ϕ. We denote $\mathcal{L}^* = \phi\mathcal{L}$, $\mathcal{C}_{\mathrm{he}}^* = \phi\mathcal{C}_{\mathrm{he}}$ and $G^* = \phi G$ the coefficients per membrane area. The generator is used in series with a second membrane of hydrodynamic resistivity $R_h^* = 1/\mathcal{L}_h^*$ per membrane area and an area $\mathcal{A}_h$. The pressure drop ΔP is applied to the complete system. The pressure drop applied to the generator is then

$$\Delta P_{\mathrm{eff}} = \Delta P - \frac{R_h^*}{\mathcal{A}_h}Q \quad [49]$$

The generated electric current is then used in a load resistance R_L.

The transport equations are:

$$\begin{pmatrix} Q \\ I \end{pmatrix} = \mathcal{A}_{\mathrm{he}}\begin{pmatrix} \mathcal{L}^* & \mathcal{C}_{\mathrm{he}}^* \\ \mathcal{C}_{\mathrm{he}}^* & G^* \end{pmatrix}\begin{pmatrix} \Delta P - R_h^* Q/\mathcal{A}_h \\ -R_L I \end{pmatrix}. \quad [50]$$

Rearranging these equations, we have:

$$\begin{pmatrix} 1+\frac{\mathcal{A}_{\mathrm{he}}}{\mathcal{A}_h}\mathcal{L}^* R_h^* & \mathcal{A}_{\mathrm{he}}\mathcal{C}_{\mathrm{he}}^* R_L^* \\ \frac{\mathcal{A}_{\mathrm{he}}}{\mathcal{A}_h}\mathcal{C}_{\mathrm{he}}^* R_h^* & 1+\mathcal{A}_{\mathrm{he}}G^* R_L \end{pmatrix}\begin{pmatrix} Q \\ I \end{pmatrix} = \mathcal{A}_{\mathrm{he}}\begin{pmatrix} \mathcal{L}^*\Delta P \\ \mathcal{C}_{\mathrm{he}}^*\Delta P \end{pmatrix}. \quad [51]$$

Inverting the matrix, the fluxes write

$$Q = \frac{1+\mathcal{A}_{\mathrm{he}}\left(1-\frac{\Lambda_{\mathrm{he}}}{1+a/4b_{\mathrm{eff}}}\right)G^* R_L}{1+\mathcal{A}_{\mathrm{he}}G^* R_L + \frac{\mathcal{A}_{\mathrm{he}}}{\mathcal{A}_h}\mathcal{L}^* R_h^* + \frac{\mathcal{A}_{\mathrm{he}}^2}{\mathcal{A}_h}\left(1-\frac{\Lambda_{\mathrm{he}}}{1+a/4b_{\mathrm{eff}}}\right)G^* R_L \mathcal{L}^* R_h^*}\mathcal{A}_{\mathrm{he}}\mathcal{L}^*\Delta P \quad [52]$$

$$I = \frac{1}{1+\mathcal{A}_{\mathrm{he}}G^* R_L + \frac{\mathcal{A}_{\mathrm{he}}}{\mathcal{A}_h}\mathcal{L}^* R_h^* + \frac{\mathcal{A}_{\mathrm{he}}^2}{\mathcal{A}_h}\left(1-\frac{\Lambda_{\mathrm{he}}}{1+a/4b_{\mathrm{eff}}}\right)G^* R_L \mathcal{L}^* R_h^*}\mathcal{A}_{\mathrm{he}}\mathcal{C}_{\mathrm{he}}^*\Delta P = \mathcal{I}I_{\max} \quad [53]$$

The electric power writes

$$\mathcal{P}_{\mathrm{e}}(R_L, R_h^*) = R_L I^2 = \frac{\mathcal{A}_{\mathrm{he}}\frac{\Lambda_{\mathrm{he}}}{1+a/4b_{\mathrm{eff}}}G^* R_L}{\left[1+\mathcal{A}_{\mathrm{he}}G^* R_L + \frac{\mathcal{A}_{\mathrm{he}}}{\mathcal{A}_h}\mathcal{L}^* R_h^* + \frac{\mathcal{A}_{\mathrm{he}}^2}{\mathcal{A}_h}\left(1-\frac{\Lambda_{\mathrm{he}}}{1+a/4b_{\mathrm{eff}}}\right)G^* R_L \mathcal{L}^* R_h^*\right]^2}\mathcal{A}_{\mathrm{he}}\mathcal{L}^*(\Delta P)^2 \quad [54]$$

which is maximal for

$$\mathcal{A}_{\rm he}G^*R_L = \frac{1+\frac{\mathcal{A}_{\rm he}}{\mathcal{A}_h}\mathcal{L}^*R_h^*}{1+\frac{\mathcal{A}_{\rm he}}{\mathcal{A}_h}\left(1-\frac{\Lambda_{\rm he}}{1+a/4b_{\rm eff}}\right)\mathcal{L}^*R_h^*} \tag{55}$$

Its maximal value is

$$\tilde{\mathcal{P}}_{\rm e}(R_h^*) = \frac{\frac{\Lambda_{\rm he}}{1+a/4b_{\rm eff}}}{\left[1+\frac{\mathcal{A}_{\rm he}}{\mathcal{A}_h}\mathcal{L}^*R_h^*\right]\left[1+\frac{\mathcal{A}_{\rm he}}{\mathcal{A}_h}\left(1-\frac{\Lambda_{\rm he}}{1+a/4b_{\rm eff}}\right)\mathcal{L}^*R_h^*\right]}\frac{\mathcal{A}_{\rm he}\mathcal{L}^*(\Delta P)^2}{4} \tag{56}$$

Dividing that the area of both membranes, the power per area of membrane is

$$\tilde{\mathcal{P}_{\rm e}}^* = \frac{\tilde{\mathcal{P}}_{\rm e}}{\mathcal{A}_{\rm he}+\mathcal{A}_h} = \frac{\frac{\Lambda_{\rm he}}{1+a/4b_{\rm eff}}}{\left[1+\frac{\mathcal{A}_{\rm he}}{\mathcal{A}_h}\mathcal{L}^*R_h^*\right]\left[1+\frac{\mathcal{A}_{\rm he}}{\mathcal{A}_h}\left(1-\frac{\Lambda_{\rm he}}{1+a/4b_{\rm eff}}\right)\mathcal{L}^*R_h^*\right]}\frac{\mathcal{L}^*(\Delta P)^2}{4\left(1+\frac{\mathcal{A}_h}{\mathcal{A}_{\rm he}}\right)} \tag{57}$$

The ratio $\mathcal{A}_{\rm he}/\mathcal{A}_h$ is a parameter which can be optimised. The optimal value for this ratio is obtained from the root of a polynomial of third order.

In practice, we are interested in situations where $\mathcal{L}^*R_h^* \gg 1$ and $1-\frac{\Lambda_{\rm he}}{1+a/4b_{\rm eff}} \ll 1$. We can identify the two following regimes:

- If $\mathcal{A}_{\rm he}/\mathcal{A}_h \ll 1/\mathcal{L}^*R_h^* \ll 1$ then $\tilde{\mathcal{P}_{\rm e}}^* \propto \mathcal{A}_{\rm he}/\mathcal{A}_h$.
- If $1/\mathcal{L}^*R_h^* \ll \mathcal{A}_{\rm he}/\mathcal{A}_h \ll \min[1/\mathcal{L}^*R_h^*(1-\frac{\Lambda_{\rm he}}{1+a/4b_{\rm eff}}),1]$ then $\tilde{\mathcal{P}_{\rm e}}^* \propto 1$.
- If $1/\mathcal{L}^*R_h^* \ll \min[1/\mathcal{L}^*R_h^*(1-\frac{\Lambda_{\rm he}}{1+a/4b_{\rm eff}}),1] \ll \mathcal{A}_{\rm he}/\mathcal{A}_h \ll \max[1/\mathcal{L}^*R_h^*(1-\frac{\Lambda_{\rm he}}{1+a/4b_{\rm eff}}),1]$ then $\tilde{\mathcal{P}_{\rm e}}^* \propto \mathcal{A}_h/\mathcal{A}_{\rm he}$.

Thus, the optimal value for $\mathcal{A}_{\rm he}/\mathcal{A}_h$ is expected to be between $1/\mathcal{L}^*R_h^*$ and $1/B = 1/\max[1/\mathcal{L}^*R_h^*(1-\frac{\Lambda_{\rm he}}{1+a/4b_{\rm eff}}),1]$. In such a case, Eq. (60) simplifies to:

$$\tilde{\mathcal{P}_{\rm e}}^* \approx \frac{\frac{\mathcal{A}_{\rm he}}{\mathcal{A}_h}}{\left[1+\frac{\mathcal{A}_{\rm he}}{\mathcal{A}_h}\mathcal{L}^*R_h^*\right]\left[1+\frac{\mathcal{A}_{\rm he}}{\mathcal{A}_h}B\right]}\frac{\mathcal{L}^*(\Delta P)^2}{4} \tag{58}$$

Therefore, the maximal power is obtained for:

$$\max[\mathcal{L}^*R_h^*(1-\frac{\Lambda_{\rm he}}{1+a/4b_{\rm eff}}),1] \ll \frac{\mathcal{A}_h}{\mathcal{A}_{\rm he}} \ll \mathcal{L}^*R_h^* \tag{59}$$

and reaches

$$\mathcal{P}_{\rm e}^{*\max} \approx \frac{\mathcal{L}_h^*(\Delta P)^2}{4} \tag{60}$$

Here, the takeaway is that the generator's permeance should be much larger than the permeance of the external membrane in order to produce the maximal power. However, if the generator's hydrodynamic resistance becomes too small, it becomes "transparent" and does not produce any power. These constraints can be managed thanks to the special effect of the hydro-electronic coupling which both dominates the friction and make the conversion. Interestingly, the external load resistance should be much larger than the generator's internal electric resistance in order to increase the generated power. The hydraulic power writes

$$\mathcal{P}_{\rm h} = Q\Delta P = \frac{1+\mathcal{A}_{\rm he}\left(1-\frac{\Lambda_{\rm he}}{1+a/4b_{\rm eff}}\right)G^*R_L}{1+\mathcal{A}_{\rm he}G^*R_L+\frac{\mathcal{A}_{\rm he}}{\mathcal{A}_h}\mathcal{L}^*R_h^*+\frac{\mathcal{A}_{\rm he}^2}{\mathcal{A}_h}\left(1-\frac{\Lambda_{\rm he}}{1+a/4b_{\rm eff}}\right)G^*R_L\mathcal{L}^*R_h^*}\mathcal{A}_{\rm he}\mathcal{L}^*(\Delta P)^2 \tag{61}$$

Thus, the efficiency is

$$\gamma = \frac{\mathcal{A}_{\rm he}\frac{\Lambda_{\rm he}}{1+a/4b_{\rm eff}}G^*R_L}{\left[1+\mathcal{A}_{\rm he}\left(1-\frac{\Lambda_{\rm he}}{1+a/4b_{\rm eff}}\right)G^*R_L\right]\left[1+\mathcal{A}_{\rm he}G^*R_L+\frac{\mathcal{A}_{\rm he}}{\mathcal{A}_h}\mathcal{L}^*R_h^*+\frac{\mathcal{A}_{\rm he}^2}{\mathcal{A}_h}\left(1-\frac{\Lambda_{\rm he}}{1+a/4b_{\rm eff}}\right)G^*R_L\mathcal{L}^*R_h^*\right]} \tag{62}$$

This efficiency could be optimized. However, it is more interesting for applications to work at maximal power. Thus, focusing on the previous ansatz for R_L and $\mathcal{A}_{\rm he}/\mathcal{A}_h$, the effective efficiency is

$$\gamma_{\rm eff} \approx \frac{1}{2} \tag{63}$$

This is an upper boundary condition which is valid for a perfect conversion $\frac{\Lambda_{\rm he}}{1+a/4b_{\rm eff}} = 1$.

C. Osmotic energy harvesting with a selective membrane. We consider the case of osmotic energy harvesting in pressure retarded osmosis (PRO). We use in series a first selective membrane of permeance $\mathcal{L}_h^*$ and a hydronic generator membrane with figure of merit $Z \gg 1$. A good selective membrane would have a permeance around $\mathcal{L}_h^* \approx 2$ L/m^3.h.bar. Thanks to the selective membrane, the separation of fresh and sea water induces an osmotic pressure $\Delta\Pi \approx 30$ bar. Thus, after optimizing of the ratio of membrane areas and external load resistance, one can expect to generate an electric power

$$\mathcal{P}_\mathrm{e}^{*\max} \approx \frac{\mathcal{L}_h^*(\Delta\Pi)^2}{4} \approx 15\ \mathrm{W/m^2} \qquad [64]$$

with an efficiency $\gamma_\mathrm{eff} \approx 50\%$.

References

1. ND Lang, W Kohn, Theory of Metal Surfaces: Charge Density and Surface Energy. *Phys. Rev. B* **1**, 4555–4568 (1970).
2. N Kavokine, ML Bocquet, L Bocquet, Fluctuation-induced quantum friction in nanoscale water flows. *Nature* **602**, 84–90 (2022).
3. B Coquinot, L Bocquet, N Kavokine, Quantum Feedback at the Solid-Liquid Interface: Flow-Induced Electronic Current and Its Negative Contribution to Friction. *Phys. Rev. X* **13**, 011019 (2023).
4. M Fukuchi, The Energy Band Structure of the Metallic Copper: The Orthogonalized Plane Wave Method. *Prog. Theor. Phys.* **16**, 222–230 (1956).
5. WA Harrison, Band Structure of Aluminum. *Phys. Rev.* **118**, 1182–1189 (1960).
6. YJ Lim, K Goh, M Kurihara, R Wang, Seawater desalination by reverse osmosis: Current development and future challenges in membrane fabrication – A review. *J. Membr. Sci.* **629**, 119292 (2021).
7. E Secchi, et al., Massive radius-dependent flow slippage in carbon nanotubes. *Nature* **537**, 210–213 (2016).
8. S Marbach, L Bocquet, Osmosis, from molecular insights to large-scale applications. *Chem. Soc. Rev.* **48**, 3102–3144 (2019).
